# Spatial distribution of human visceral leishmaniasis cases in Cametá, Pará, Eastern Amazon, Brazil

**DOI:** 10.1590/0037-8682-0220-2021

**Published:** 2021-11-15

**Authors:** Claudia do Socorro Carvalho Miranda, Jorge Bonito, Rafael Aleixo Coelho de Oliveira, Juan Andrade Guedes, Conceição Santos, João Sérgio de Sousa Oliveira, Tainara Garcia Filgueiras, Francisco de Assis Oliveira, Tainã Garcia Filgueiras, Nelson Veiga Gonçalves

**Affiliations:** 1 Universidade do Estado do Pará, Laboratório de Epidemiologia e Geoprocessamento da Amazônia, Belém, PA, Brasil.; 2 Escola Superior da Amazônia, Belém, PA, Brasil.; 3 Universidade de Évora, Escola de Ciências Sociais, Évora, Portugal.; 4 Universidade de Aveiro, Centro de Investigação Didática e Tecnologia na Formação de Formadores, Aveiro, Portugal.; 5 Universidade do Porto, Laboratório Associado para Química Verde, Rede de Química e Tecnologia, Porto, Portugal. Universidade de Trás-os-Montes e Alto Douro, Centro de Investigação em Tecnologias Agroambientais e Biológicas da Vila Real, Portugal.; 6 Universidade Federal Rural da Amazônia, Laboratório de Ciências do Solo, Belém, PA, Brasil.; 7 Universidade do Estado do Pará, Centro de Ciências Biológicas e da Saúde, Departamento de Saúde Comunitária, Belém, PA, Brasil.

**Keywords:** Environmental health, Epidemiology, Leishmaniasis, Spatial analysis

## Abstract

**INTRODUCTION:**

Human visceral leishmaniasis is considered a major public health problem in the municipality of Cametá, Pará, Brazil.

**METHODS:**

This ecological study was conducted using data obtained from official Brazilian agencies. Spatial analysis of the variables was performed using the kernel interpolation technique and the bivariate Global Moran’s Index.

**RESULTS:**

The spatial distribution of the disease was not homogeneous, and it was associated with socio-environmental risk factors and public policies.

**CONCLUSIONS:**

Different relationships were observed between the variables studied and disease occurrence.

Human visceral leishmaniasis (HVL) is a zoonosis caused by the protozoan *Leishmania chagasi* (Ord. Trypanosomatidae), which is transmitted by infected female phlebotomine sandflies (Ord. Diptera). In Brazil, the main species responsible for HVL transmission is *Lutzomyia longigalpis* ^1^. The main reservoirs of the disease are dogs in the domestic environment and marsupials in the wild environment^1^ ^,^ ^2^.

HVL affects 76 countries, 12 of which are in the Americas^2^. Brazil accounted for 90% of the cases recorded for Latin America in 2017; for 2003-2018, more than 51,000 human cases of the disease were confirmed, with an average incidence of 1.7 cases/100,000 inhabitants, a variation of 1.4-2.1 and a lethality of 7.2% in the Brazilian territory^3^ ^,^ ^4^.

In the state of Pará, the dynamics of deforestation and unsustainable land use and occupation, historically developed using the macrodevelopmentalist model chosen for the Amazon^5^ ^-^ ^7^ have contributed to vulnerability among human populations to various infectious diseases. Among them, HVL occupies a prominent place in the epidemiological scenario because of its relationship with environmental degradation.

In this context, the Cametá municipality in northeastern Pará state is undergoing an intense deforestation process due to different human actions, including logging, agriculture and ranching, disorganized urbanization, and mining. In recent years, the municipality has also seen an increase in the number of HVL cases, with the state’s third-largest case count^3^ ^,^ ^8^ ^,^ ^9^. There is evidence of socioenvironmental causes of the disease related to precarious public health policies, especially in terms of coverage with Basic Health Units (BHU).

To produce an epidemiological scenario that can consider the possible correlations between the occurrence of HVL and the socioenvironmental conditions and public policies of Brazilian territories, geotechnologies have been used in recent decades as fundamental tools in the process. These make it possible to describe the geographic distribution of the disease and its related health risks. Thus, this study sought to analyze the spatial distribution of human visceral leishmaniosis and its relationship with environmental variables and public policies in Cametá municipality, state Pará, Brazil. 

The population of this ecological study consisted of 210 notified and confirmed cases of HVL in the municipality of Cametá, State of Pará, from 2007 to 2016. The municipality has an area of 3.081,367 km² that is divided into nine administrative districts with an estimated population of 139,364 inhabitants^10^.

The epidemiological data (sex, age, ethnicity, education, and region) were obtained from the Disease Notification Information System of the Municipal Health Departament in Cametá and the State Department of Health of Pará. Cartographic data on municipal boundaries from the SIRGAS 2000 projection and demographic (population) data were obtained from the Census 2010 database provided by the Brazilian Institute for Geography and Statistics. The environmental databases related to land use and cover (pasture, agriculture, urban area, forest, mining, secondary vegetation, deforestation, and other classes) were obtained from the Amazon Land Use and Cover Project (TerraClass2019) of the National Space Research Institute. The variables for public health policies (services provided by the BHU) were obtained from the National Health Establishment Registry.

The study used municipal administrative districts as spatial units for analysis (Cametá, Areião, Carapajó, Curuçambaba, Joana Coeli, Juaba, Moiraba, Vila do Carmo de Tocantins, and Torres do Cupijó). TabWin 36b was used for debugging the databases and removing incomplete, inconsistent, and allochthonous records. After that, three expeditions were made to the study areas to georeference the cases and identify the land use and cover types using a global positioning system to create a georeferenced database. In the descriptive and inferential analyses of the epidemiological and public health system variables, percentage calculations were performed using BioEstat 5.0, free software for Biostatistical analysis (José Ayres and others and was funded by CNPq, Brazil).

The following thematic classes were used to analyze land use and cover: urban area, occupation (rural communities and locations), agriculture, forest, pasture (clean, overgrown, and regenerated pasture), deforestation, and secondary vegetation. The other classes provided by the TerraClass project were grouped into “other classes.” The grassland vegetation formation was separated into the “non-forest” class because of its epidemiological value as a potential ecotope for HVL vectors.

The percentage of deforestation in the districts was calculated based on the sum of urban area, occupation, agriculture, pasture, deforestation, secondary vegetation, wooded savanna and “other classes,” which was divided by their total area using the Calculate Geometry tool. The geostatistical analysis of the case distribution pattern was performed with the kernel density estimation using ArcGIS 10.5 software.

The Moran Global Bivariate (I) index was employed to assess the spatial autocorrelation between areas with deforestation and those with HVL. For that purpose, the hypotheses of “inverse” (I < 0), “random” (I = 0), and “direct” (I > 0) spatial aurocorrelation were admitted, with a significance to p < .05. To perform this work, the principles of the Helsinki Declaration, the Nuremberg Code, and the norms of Resolution no. 466/12 of the National Health Council were followed, and favorable report no. 3.292.673 was received from the Research Ethics Committee of the State University of Pará.

Two hundred and ten confirmed cases of HVL were analyzed in the study area and period. A decreasing trend was observed in the number of cases of the disease, with two peaks appearing in 2008 and 2012 (Figure 1).

**FIGURE 1:**
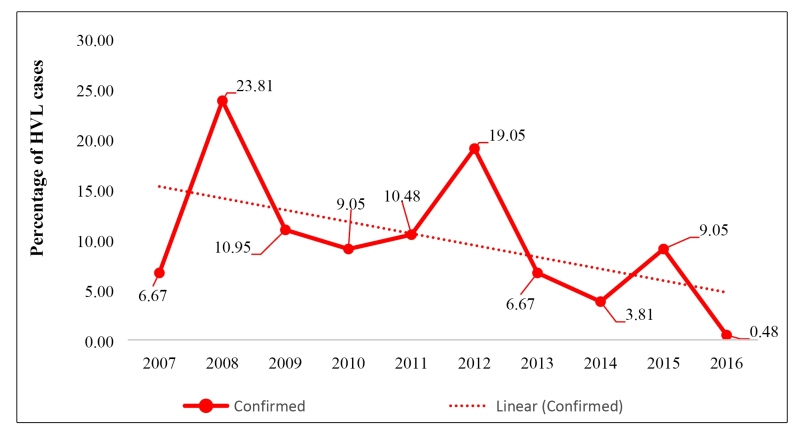
Historical series of HVL cases in Cametá, Pará, Brazil, 2007-2016.

The distribution of HVL cases for all months in the study period occurred with values close to the average (7 cases/month) except for July to September, which presented a slight increase in cases.

The highest percentage of cases occurred in individuals of the male sex (59.0%), those under 12 years of age (69.0%), those with brown-skin classification (63.3%), those younger than school age (ages 0-4 years) (58.1%), and those in rural areas (63.8%) (Table 1).

**TABLE 1:** Epidemiological profile cases of HLV in the municipality of Cametá, Pará, Brazil, 2007-2016.

Variable		N = 210	%
**Sex**	Male	124	59.0
	Female	86	41.0
**Age**	Child (≤12)	145	69.0
	Teenager (>12-17)	19	9.0
	Adult (>17-59)	43	20.5
	Elder (>59)	3	1.5
**Ethnicity**	Brown	133	63.3
	White	17	8.1
	Black	7	3.3
	Unknown	53	25.3
**Education**	Illiterate	0	0
	Basic education	34	16.2
	University education	0	0
	Not applicable	122	58.1
	Unknown	54	25.7
**Region**	Rural	134	63.8
	Urban	38	18.1
	Periurban	1	.5
	Unknown	37	17.6

In the location map for the occurrence of disease, a non-homogeneous distribution was observed, with clusters of cases in certain rural areas of the Juaba district and in the urban center of the municipality (Figure 2A).

An analysis of the spatial occurrence of HVL also showed the occurrence of high densities of cases at the municipal seat and in other areas of the Cametá district near the Cupijó River. High densities of cases were also observed in some areas of the administrative districts of Juaba, Torres de Cupijó, and Moraiba. In terms of BHU distribution as part of public policies for basic and secondary health care in the municipality, the highest coverage was seen at the municipal seat in Cametá, while the other districts had low coverage (Figure 2B).

A spatial analysis of land use and cover revealed many high levels of environmental changes, such as pasture and secondary vegetation, especially along the Cupijó, Anauerá, and Tocantins Rivers. Grassland vegetation, an ecotope for phlebotomids, was also observed, mainly along the BR - 422 highways. In these areas, a high level of HVL occurrence was observed, indicating a process of environmental vectorization (Figure 2C).

**FIGURE 2:**
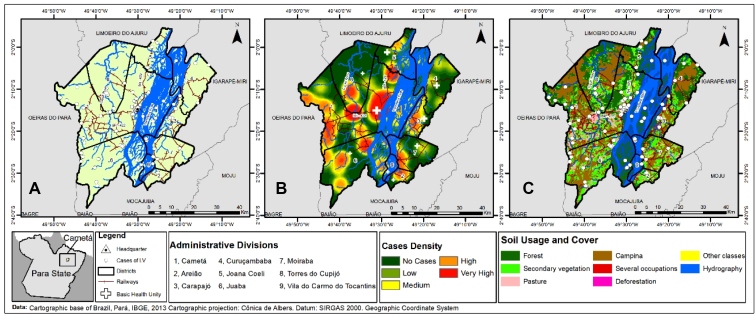
(A) Spatial distribution of HVL cases in Cametá, Pará, Brazil, 2007-2016; (B) HVL cases density and BHU spatial distribution in Cametá, Pará, Brazil, 2007-2016; (C) Spatial distribution of HVL cases and types of soil usage and cover in the Cametá, Pará, Brazil, 2007-2016. Source: EpiGeo/DSCM/CCBS/UEPA, 2019.

The Moran Global Bivariate (I) index showed significant direct spatial autocorrelations between areas with HVL cases and those with deforestation, with positive indices (I > 0) and p = .0001, in the nine districts of the municipality. These autocorrelations were strong in Cametá (I = .7891) and Juaba (I = .6075).

HVL is a public health problem in the municipality of Cametá because of the intense level of transmission in that territory, which makes it a priority area for measures to follow up and control the disease through actions in epidemiological and environmental surveillance^1^ ^,^ ^3^ ^,^ ^8^ ^,^ ^9^. The complex endemicity of the disease in this municipality is related to socio-environmental and demographic characteristics that favor its transmission, such as an increase in inadequate housing conditions, human settlements in vulnerable areas, marked deforestation, and migratory flow over the last few decades^8^ ^,^ ^9^. The continuation of these conditions in the municipality has negative implications for fulfilling the health ministry’s goals for controlling HVL.

The higher occurrence of HVL in the months of July to September may be related to reproductive dynamics among phlebotomids and the incubation period for the disease. Thus, at the end of the rainy season in the Amazon in May and June, due to environmental conditions (temperature, rainfall, and humidity), there is an increase in the population density of these vectors, enabling a higher probability of disease transmission^11^ ^,^ ^12^ ^,^ ^13^. The incubation period indicates a higher number of notifications in the months above, since it occurs 2 to 6 months after infection^2^.

The epidemiological profile of men, children, and brown-skinned individuals observed in this study are recurrent in other Amazon territories, indicating the association of different behavioral, immunological, and populational factors related to those variables, including greater exposure to disease vectors, immature immunological systems (aggravated by nutritional deficiencies), and the ethnic origins of the Amazon population^14^ ^,^ ^15^.

The predominance of cases with education as “not applicable” may be related to children in the 0-to-4-year age range. This possibility points towards the establishment of a household and household peri-household transmission circuit for HVL since the children are not yet in school. Thus, a socioenvironmental production of the disease was observed in the municipality based on the logic of socially inequitable exclusion, reflected in the precarious public health policies that mainly affect the protection of children’s health.

Spatial analyses showed a greater density of cases in the Cametá district, possibly because of its higher population (urban and rural) and greater availability of health services because it is a municipal seat. However, the low number of cases in certain areas of other districts with lower coverage indicates a possible epidemiological silence, suggesting that the socioeconomic conditions of its population determine differences in access to health services. Thus, in those areas in the municipality that have major social vulnerabilities, health care provision does not occur as a fundamental right of citizenship as determined by the Health Unic System, since in those areas, persons with the greatest need have the lowest level of access to health services. 

Spatial analysis of land use and cover types showed a high percentage of deforested areas because of human activities in the municipality, including agriculture, ranching, and mining in the grassland areas^8^ ^,^ ^9^. Additionally, the disorderly urban expansion has established new socio-spatial configurations, with human settlements located along the Cupijó, Anauerá, and Tocantins Rivers, which over the last few decades have undergone socioenvironmental degradation that leads to circuits for transmitting the disease based on poverty-disease spatial dependence.

The very high density of cases along the BR-422 highway and its feeder roads suggests that these routes are a means for the spatial vectorization of HVL with dissemination through a transmission chain. This fact, associated with the precarious sanitation infrastructure in population centers located along those epidemiological corridors, leads to a major public health problem for the municipality^12^.

The direct and strong spatial autocorrelation observed in some areas with a large number of cases of the disease and a high percentage of deforestation in the Cametá and Juaba districts highlights the negative impacts resulting from the cumulative pressure of anthropic relations practiced in the municipality and their effects on the environment with harmful consequences on the health of human populations, a direct antithesis to sustainable development^5^ ^,^ ^7^.

Considering the above, we recommend the expansion of measures for HVL control and follow-up, especially the active search for cases and performance of laboratory examinations. These should be accompanied by education, work, housing, and basic sanitation conditions in an effort to promote social equity and environmental respect.

## References

[1] Brasil. Ministério da Saúde (2019). Guia de Vigilância em Saúde.

[2] World Health Organization (2018). Status of endemicity of visceral leishmaniasis wordwide.

[3] Brasil. Ministério da Saúde (2020). Leishmaniose visceral - casos confirmados notificados no sistema de informação de agravos de notificação - Paraná.

[4] Silva RA, Alves RV, Júnior FEFL, Costa JNG, Donato LE, Gomes MLS (2019). Leishmaniose visceral. Boletim Epidemiológico. Vigilância em saúde no Brasil 2003-2019.

[5] Oliveira AP, Aguiar ES, Pontes AN (2020). Neglected tropical diseases and socio-environmental vulnerabilities in Amazonian capitals. Research, Society and Development.

[6] Fearnside PM, Laurance WF (2012). Infraestrutura na Amazônia: as lições dos planos plurianuais. Caderno CRH.

[7] Ellwanger JH, Kulmann-Leal B, Kaminski VL, Valverde-Villegas JM, Veiga ABGD, Spilki FR (2020). Beyond diversity loss and climate change: Impacts of Amazon deforestation on infectious diseases and public health. An Acad Bras Cienc.

[8] Souza AAA, Campos MB, Barata IR, Pinheiro SMB, Silva GM, Lima JA (2008). Flebotomíneos de área endêmica de Leishmaniose visceral de Cametá - Pará, Mesorregião do Nordeste Paraense. Rev Soc Bras Med Trop.

[9] Miranda BO Silva LHSE, Viana JH (2019). Estudo epidemiológico de casos de leishmaniose visceral no município de Cametá - Pará.

[10] Instituto Brasileiro de Geografia e Estatística (IBGE) Cametá.

[11] Rutledge LC, Ellenwood DA (1975). Production of phlebotomine sandflies on the open forest floor in Panama: the species complement. Environmental Entomology.

[12] Chagas AP, Soares DC, Sousa GCR, Viana RB, Rebelo JMM, Garcez LM (2016). Ecological aspects of phlebotomine sand flies in foci of leishmaniasis in the eastern Amazon, Pará State, Brazil. Revista Pan-Amazônica de Saúde.

[13] Reis LL, Balieiro AAS, Fonseca FR, Gonçalves MJF (2019). Visceral leishmaniasis and its relationship with climate and environmental factors in the State of Tocantins, Brazil, from 2007 to 2014. Cadernos de Saúde Pública.

[14] Barbosa IR, Costa ICC (2013). Clinical and epidemiological aspects of visceral leishmaniasis in children up to 15 years of age in Rio Grande do Norte state, Brazil. Sciencia Médica.

[15] Cavalcante IJM, Vale MR (2014). Epidemiological aspects of visceral leishmaniasis (kala-azar) in Ceará in the period 2007 to 2011. Rev Bras Epid.

